# Absolute volume of the rectum and AUC from rectal DVH between 25Gy and 50Gy predict acute gastrointestinal toxicity with IG-IMRT in prostate cancer

**DOI:** 10.1186/s13014-016-0721-8

**Published:** 2016-11-04

**Authors:** Céline Mirjolet, Paul M. Walker, Mélanie Gauthier, Cécile Dalban, Suzanne Naudy, Frédéric Mazoyer, Etienne Martin, Philippe Maingon, Gilles Créhange

**Affiliations:** 1 Department of Radiation Oncology, Centre Georges Francois Leclerc, 1, rue du Pr Marion, 21049 Dijon, France; 2 Medical Imaging Group, Laboratory of Electronics, Computer Science and Imaging, (Le2I), CNRS 6306, University of Burgundy, 21000 Dijon, France; 3 Department of Biostatistics, Centre Georges Francois Leclerc, 21049 Dijon, France

**Keywords:** Prostate cancer, Acute rectal toxicity predictive factor, Radiotherapy

## Abstract

**Background:**

To determine whether dose/volume specific endpoints (DVSE) or Area under the rectal DVH curve (rAUC) better predict acute gastrointestinal (GI) toxicity in prostate cancer patients treated with IMRT in the era of daily image guidance (IG-IMRT).

**Methods:**

A set of DVSE was recorded from V25 to V75 (increments of 5Gy) (both in % and in cc) for 180 men. The rAUC was calculated for doses ranging between 25Gy and 50Gy (rAUC_25–50_). Univariate and multivariate logistic regressions were performed to determine the relationship between DVSE or rAUC_25–50_ and the appearance of any acute GI toxicity.

**Results:**

The rates of acute grade 1 (G1), G2 and G3 GI toxicities were 53.3 %, 10.6 % and 1.1 %, respectively. No G4+ toxicity was observed.

Rectal V25 to V75 expressed in % were not predictive of G ≥ 1 GI toxicity (p ≥ 0.12) whereas rectal V25 to V50 expressed in cc did correlate with GI toxicity G ≥ 1 (p ≤ 0.04). rAUC_25–50_ expressed in cc. Gy correlated significantly with the occurrence of any acute GI toxicity G ≥ 1 (*p* = 0.027).

**Conclusions:**

The absolute volume of the rectum between 25Gy and 50Gy and rAUC_25–50_ could significantly predict any acute rectal toxicity in prostate cancer patients treated with daily IG-IMRT.

## Background

Intensity Modulated Radiation Therapy (IMRT) with daily image guidance of soft tissues in patients with localized prostate cancer (PCa) has been shown to improve biochemical control and to reduce rectal toxicities compared with 3-dimensional conformal radiation therapy (3D-CRT) [1–3]. The risk of normal tissue complications is typically evaluated from the amount of tissue exposed to a given dose (namely, dose-volume specific endpoint (DVSE)). For 3D-CRT, DVSE have been demonstrated to be reliable and reproducible for predicting acute and late toxicity according to Radiation Therapy Oncology Group (RTOG) scoring criteria [4]. Several dose-constraint guidelines, based on post-hoc analyses of rectal toxicity and its correlation with each DVSE, have been established to determine what relative volume of rectum (in %) can safely receive high doses [5–8].

As for image-guided IMRT (IG-IMRT), there are no robust, reproducible data in the literature to indicate which DVSE are useful for a more accurate prediction of acute toxicity. Guidelines used by radiation clinicians were based on late toxicities and came mostly from 3D conformal radiotherapy [4]. Regarding the lower rates of rectal toxicity observed with IG-IMRT compared with 3D-CRT [9], standard DVSE developed from 3D-CRT results could be irrelevant. Hence, new tools are needed for IG-IMRT to improve predictions for any grade of acute toxicity. The purpose of this study was to determine a new type of dose/volume parameter to predict any acute GI toxicity with daily IG-IMRT. This new parameter was determined by studying a set of several DVSE expressed in % and in cc. As the shape of the entire rectal DVH curve from low to high doses cannot be reflected by one single DVSE or even several DVSE, we also investigated the area under the rectum DVH curve (rAUC) as a challenger for predicting acute GI toxicity.

## Methods

### Selection of patients

We selected 180 men with at least eight visits for toxicity evaluations and with available DVH data. All of the men had localized PCa treated with daily IG-IMRT with curative intent.

Characteristics of patients are summarized in Table 1.

**Table 1:** Characteristics of patients and treatments

	All patients
	*N* = 180
Age (median [range]) years	70.5 [49.6–84.9]
T stage-n (%)	
T1c	57 (31.7 %)
T2a	31 (17.2 %)
T2b	37 (20.6 %)
T2c	19 (10.6 %)
T3a	30 (16.7 %)
T3b	6 (3.3 %)
PSA (ng/ml) (median [range])	10 [0.8–99]
Gleason score-n (%)	
≤ 6	103 (57.2 %)
7	64 (35.6 %)
≥ 8	13 (7.2 %)
Risk groups-n (%)	
Low risk	48 (26.7 %)
Intermediate risk	68 (37.8 %)
High risk	64 (35.6 %)
Hormone therapy-n (%)	
Neoadjuvant	50 (27.8 %)
Concomitant	69 (38.3 %)
Adjuvant	70 (38.9 %)
Radiotherapy: prostate dose (2Gy/fx^a^)	78 [70–80]
Median [range]	
TURP^b^-n (%)	
Yes	35 (19.7 %)
No	143 (80.3 %)
Missing	2 (1.1 %)

^a^ fraction; ^b^ transurethral resection of the prostate

### IMRT

All patients first underwent a planning CT scan with 2.5 mm slice thickness in the supine position with knee and ankle supports. A rectal enema was given before the CT for each patient. They were asked to maintain the same degree of bladder filling during the simulation and treatment sessions. Critical normal-tissue structures were outlined by a radiation oncologist on each axial CT image. The rectum was defined as a cylindrical structure around the outer rectal wall and contoured from the ischial tuberosities to the rectosigmoid junction, identified in accordance with international guidelines by the level at which the GI tract narrows and diverges anteriorly from the rectum [4, 10].

Intensity was modulated by dynamic multileaf collimation using the sliding window technique, as previously described by our group in this journal [10, 11]. Patients who underwent whole pelvic radiotherapy were excluded from this study. The median dose prescribed to the prostate PTV was 78Gy [74–80] at 2Gy per fraction and five fractions per week.

### IGRT

Daily on-line repositioning based on soft-tissues was performed for all the patients using either kV Cone Beam Computed Tomography or a 3D ultrasound system as described in detail elsewhere [11, 12].

### Dose/volume modeling for rectal toxicity

A set of standard DVSEs was tested: the volume of the rectum receiving from 25Gy to 75Gy (V25 to V75), expressed in percentages (%) and in cubic centimeters (cc).

#### Calculation of the area under the rectum DVH curve (rAUC)

In the second step, we calculated the area under the DVH curve between 25 and 50Gy for the rectum (rAUC_25–50_).

**Fig. 1:**
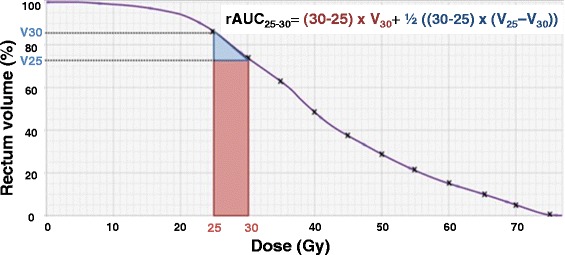
Illustration of rAUC 5Gy calculation from 25 to 30Gy (rAUC_25–30_). Legends: DVH: Dose Volume Histogram, V25 = volume of rectum receiving 25Gy; V30 = volume of rectum receiving 30Gy; rAUC_25–30_ = Area Under the Curve of rectal DHV between 25 and 30Gy

The respective rAUC, expressed in cc.Gy, were calculated every 5Gy in the following manner (Fig. 1):

$$ {\mathrm{rAUC}}_{\mathrm{doseX}\hbox{-} \mathrm{X}+5\mathrm{Gy}=}5*{\mathrm{V}}_{\mathrm{X}+5\mathrm{Gy}} + 2.5*\left({\mathrm{V}}_{\mathrm{X}\mathrm{Gy}}\hbox{-} {\mathrm{V}}_{\mathrm{X}+5\mathrm{Gy}}\right) $$

For example (Fig. 1): rAUC_25 ‐ 30_ = 5 * V30 + 2.5 * (V25 ‐ V30)

Thus, rAUC_25 ‐ 50_ = rAUC_25 ‐ 30_ + rAUC_30 ‐ 35 + r_AUC_35 ‐ 40_ + rAUC_40 ‐ 45_ + rAUC_45 ‐ 50_

#### Follow-up and toxicity evaluation

Each patient was seen in our institution every week during the radiation therapy and at 3 months and 6 months thereafter. Acute toxicity was evaluated and scored using the Common Toxicity Criteria Adverse Events scales (CTCAE) version 3.0 weekly during each week of radiotherapy and 3 months after the completion of the radiotherapy. As acute toxicities can last for 3 months, we chose to extend our evaluation to 6 months. The worse GI toxicity (diarrhea, constipation, hemorrhoids, rectal hemorrhage, anal incontinence, proctitis and anitis) grade of each patient was analyzed.

#### Statistical analyses

Mann–Whitney tests were used to determine the relationship between V25-V75, expressed in % and in cc or rAUC_25–50_ and the appearance of any acute GI toxicity (G ≥ 1).

The optimal rAUC_25–50_ cut-off value related to acute GI toxicity was determined using a ROC curve with Youden’s index. Univariate and multivariate logistic regressions were used to describe the acute GI toxicity (G ≥ 1) by estimating the Odds-Ratio and 95 % confidence interval (CI). The multivariate model included the optimal rAUC_25–50_ cut-off value and the adjustment parameters (age, transurethral resection of the prostate (TURP), hormone therapy and rectum volume). The multivariate model was internally validated using bootstrapping (170 replications).

All analyses were performed using Stata V13 software (StataCorp LP, College Station, TX). *P* values were two-tailed and considered significant when less than 0.05.

## Results

### Characterization of toxicity

We observed acute G1, G2 and G3 GI toxicity in 96 patients (53.3 %), 19 patients (10.6 %) and 2 patients (1.1 %), respectively. No G4 or G5 GI toxicity was observed. Among these toxicities, 35.8 % of patients had diarrhea (29.6 % G1 and 6.2 % G2) and 36.1 % of patients had proctitis (32.2 % G1, 3.3 % G2 and 0.6 % G3).

#### Predictors of acute GI toxicity

The median volumes of the rectum in % and in cc receiving from 25Gy to 75Gy (increments of 5Gy) are presented in Table 2.

**Table 2:** Evaluation of relationship between DVSE and acute gastrointestinal toxicity using univariate logistic regression analysis

Median [range]	All patients	Patients with acute toxicities G^b^ = 0	Patients with acute toxicities G ≥ 1	*p*-value
	*n* = 180	*n* = 63	*n* = 117	
Rectum volumes expressed in %				
V25^a^	68 [27;100]	69.6 [27;100]	67.7 [31.6;100]	0.799
V30	60.9 [24.2;100]	61.6 [24.2;100]	60.7 [25.5;100]	0.860
V35	51.9 [21;100]	51.7 [21.8;99.6]	52.1 [21;100]	0.726
V40	44 [17.6;96.7]	44.3 [19.7;85.3]	43.9 [17.6;96.7]	0.638
V45	35.2 [13.1;82.2]	37.1 [15.5;62.8]	34.8 [13.1;82.2]	0.704
V50	27.8 [8;74.6]	28.3 [9.3;52.9]	27.7 [8;74.6]	0.766
V55	22.4 [5.4;51.2]	23 [5.4;43.6]	22.3 [6.1;51.2]	0.875
V60	17.2 [2.7;40.8]	17.6 [2.7;35.1]	17 [3.4;40.8]	0.918
V65	11.8 [1;29.5]	12.6 [1;29.5]	11.5 [1.2;26.5]	0.582
V70	7.3 [0;23.1]	7.8 [0;23.1]	6.7 [0;19.8]	0.217
V75	0.9 [0;11.4]	1.5 [0;11.4]	0.7 [0;9.1]	0.124
Rectum volumes expressed in cc				
V25	53.5 [20.1;223]	48.8 [20.1;114]	57 [20.6;223]	0.039
V30	47.5 [19.5;180.2]	42.8 [19.5;99.8]	50.9 [19.8;180.2]	0.045
V35	41.7 [16.7;150]	37.8 [16.7;86]	42.6 [17.4;150]	0.035
V40	36.1 [11;120.8]	31.9 [11;79]	37.4 [13.9;120.8]	0.025
V45	29.8 [8;101.9]	25.8 [8;69.1]	31.6 [10.2;101.9]	0.018
V50	24.1 [5.9;77]	21.1 [5.9;58.1]	25 [7.6;77]	0.035
V55	19 [4.4;56.4]	17.9 [4.4;47.8]	19.7 [5.8;56.4]	0.069
V60	14.6 [2.4;43.6]	13.1 [2.4;36.3]	15.2 [3.1;43.6]	0.17
V65	10.2 [0.9;36.8]	9.9 [0.9;28.7]	11 [1.3;36.8]	0.387
V70	5.7 [0;29.5]	5.8 [0;20.2]	5.7 [0;29.5]	0.853
V75	0.8 [0;16.2]	0.9 [0;8]	0.6 [0;16.2]	0.232
rAUC_25–50 (cc. Gy)_^c^	972.5 [388.5;3305.3]	835.7 [394.3;2008.8]	1002.4 [388.5;3305.3]	0.028

^a^ Vx = volume of rectum receiving xGy; ^b^ Grade using CTC-AE V3.0 validated scale; ^c^ Area Under the Curve of rectal DHV between 25 and 50Gy

In the univariate analysis of the entire patient population, we found no relationship between any rectal volume parameters expressed in % and any acute GI toxicity ≥ grade1 (*p* from 0.12 to 0.92) (Table 2).

Conversely, when expressed in cc, all rectal volumes from V25 to V50 correlated significantly with acute GI toxicity G ≥ 1 (p from 0.018 to 0.045). Beyond 50Gy, no relationship was found between the volume of rectum expressed in cc and acute GI toxicity (from 55Gy to 75Gy, *p*-values ranged between 0.069 and 0.853) (Table 2).

The rAUC_25–50_ calculated using the rectum volume expressed in cc correlated with any grade ≥1 acute GI toxicity (*p* = 0.028) (Table 2) while the rAUC_25–50_ calculated using rectum volume expressed in % did not correlate with any acute GI toxicity (data not shown).

Multivariate logistic regression, which included the variables age, hormone therapy, TURP and rectum volume (cc), was used. Among these variables, only rectum volume expressed in cc correlated significantly with acute GI toxicity (*p* = 0.041). A Liu/Youden cutting method showed that patients with a rAUC_25–50_ > 794 cc.Gy were more likely to develop acute GI toxicity with IG-IMRT (*p* = 0.020, [95 % CI: 1.16–5.46]) (Table 3). These results were validated by a bootstrapping method using 170 replications (*p* = 0.019; [95 % CI: 1.16–5.42]).

**Table 3:** Optimal rAUC_25–50_ cut-off value related to acute GI toxicity Grade ≥ 1 determined using a ROC curve (multivariate logistic regression)

	Univariate analysis				Multivariate analysis				Bootstrapping (170 rep)	
	Acute GI^a^ toxicity G ≥ 1 / N	OR^c^	95 % CI^d^	*p*-value	Acute GI toxicity G ≥ 1 / N	OR	95 % CI	*p*-value	95 % CI	*p*-value
					115/178					
rAUC_25-50_^b^ rectum										
Liu/Youden cutting methods										
< =794 cc.Gy	29/59	1		0.002	28 / 58	1		0.020		0.019
> 794 cc.Gy	88/121	2.76	[1.44;5.28]		87 / 120	2.51	[1.16;5.46]		[1.16;5.42]	
Adjustment variables										
Age										
< 70 years	57/82	1		0.247	57 / 82	1		0.233		0.226
> = 70 years	60/ 98	0.69	[0.37;1.29]		58 / 96	0.67	[0.35;1.29]		[0.35;1.28]	
TURP^e^										
No	95/143	1		0.305	95 / 143	1		0.423		0.459
Yes	20/35	0.67	[0.32;1.43]		20 / 35	0.72	[0.33;1.59]		[0.31;1.7]	
HT^f^										
No	65/104	1		0.411	64 / 103	1		0.757		0.755
Yes	52/76	1.3	[0.7;2.43]		51 / 75	1.11	[0.57;2.16]		[0.57;2.15]	
Rectum Volume (cc) per unit		1		0.041		1		0.558		0.570
		1.01	[1;1.02]			1	[0.99;1.01]		[0.99;1.01]	

^a^gastrointestinal; ^b^Area Under the Curve of rectal DHV between 25 and 50Gy, ^c^odds ratio; ^d^95 % confidence interval;^e^ transurethral resection of the prostate; ^f^hormonotherapy

## Discussion

One of the major limits of DVSE is that the DVH curve can reach V70 by different paths, meaning that doses delivered before or beyond this specific endpoint might differ considerably for the same V70. Given this, for the same volume of rectum receiving a high dose, one patient may have a greater rectal volume irradiated at lower doses while another may have his rectum spared when evaluated according to the planning CT. For this reason, the QUANTEC recommended several DVSE in the context of dose escalation delivered using 3D conformational radiotherapy [4]. Recently, like us, Pederson et al. found a lack of any correlation between standard DVSE criteria and late GI or GU (genitourinary) toxicities, evaluated using RTOG and CTCAE V3.0 scales, induced by IMRT [13]. The authors suggested adapting rectum DVH, which correlated with late toxicities induced by IMRT. A new parameter related to acute toxicity has yet to be developed.

A number of preliminary clinical studies on high-dose IMRT and/or high-dose IGRT, like ours, reported very low rates of acute GI toxicity. In routine practice with IG-IMRT, acute toxicity is much more frequent than late toxicity and most patients have grade 1 or 2 toxicity only, suggesting that it would be better to figure out what dose/volume parameters best predict any acute GI toxicity (*n* = 117 in our series) rather than severe GI toxicity only. Although acute toxicities were prospectively recorded in the follow-up of all of the patients, one limitation of our study arises from the retrospective design, with drawbacks related to the post hoc scoring of toxicity using version 3 of the CTC scale.

Even though toxicity was prospectively scored, one drawback of our retrospective analyses concerns the major differences in scoring systems in the literature. Patient-reported outcomes may be more clinically relevant and we therefore suggest conducting a new prospective study that includes both physician- and patient-reported outcomes.

Nevertheless we found a lower rate of acute G ≥ 2 GI toxicity in our series (12 %), which was very similar to that observed by Kupelian et al. in 488 patients with daily IG-IMRT (11 % of G2 acute rectal toxicity) [14] but lower than that observed by Wortel et al. in 260 patients treated with IMRT (29 % of G ≥ 2 acute rectal toxicity) [9]. These two studies used the RTOG toxicity scoring system. In another report, Singh et al. also confirmed less severe rectal symptoms with IGRT compared with non-IGRT [2].

We believe that daily IG-IMRT with a sharper dose gradient, thanks to daily repositioning on the prostate isocenter, may ensure that high doses are only delivered to the same small areas of the rectum as is the case with brachytherapy. Our results are in keeping with this hypothesis as we found that only the absolute volume of the rectum (but not the relative volume of the rectum) between 25Gy and 50Gy correlated with acute GI toxicity when patients were treated with daily on-line IG-IMRT.

Moreover, these results suggest that intermediate doses delivered to the rectum may be more relevant than high doses with daily IG-IMRT.

## Conclusions

We have used a simple method to identify a new single parameter derived from the DVH, in contrast to several DVSE, that predicts acute GI toxicity: the rAUC_25–50_ is a user-friendly tool that can be implemented in any radiation oncology department worldwide.

We recommend that the rAUC_25–50_ of the entire rectum should not exceed 794 cc.Gy. This new predictive parameter for acute GI toxicity should be validated through a prospective study.

## Abbrevations

3D-CRT: 3-dimensional conformal radiation therapy
CTCAE: Common terminology criteria for adverse events
CTV: Clinical target volume
DVH: Dose volume histogram
DVSE: Dose/volume specific endpoints
GI: Gastrointestinal
GTV: Gross tumor volume
GU: Genito-urinary
HT: Hormonotherapy
IG-IMRT: Image-guidance intensity modulated radiation therapy
OAR: Organs at risk
PCa: Prostate cancer
PTV: Planning target volume
rAUC: Area under the rectal DVH curve
rAUC_25–50_: rAUC for doses ranging between 25Gy and 50Gy
RTOG: Radiation Therapy Oncology Group
TURP: Transurethral resection of the prostate
Vx: Volume of rectum receiving xGy
